# The Final Phases of Ovarian Aging: A Tale of Diverging Functional Trajectories

**DOI:** 10.3390/jcm14165834

**Published:** 2025-08-18

**Authors:** Stefania Bochynska, Miguel Ángel García-Pérez, Juan J. Tarín, Anna Szeliga, Blazej Meczekalski, Antonio Cano

**Affiliations:** 1 Department of Gynecological Endocrinology, Poznan University of Medical Sciences, 61-701 Poznan, Poland; stefaniabochynska@gmail.com (S.B.); annamaria.szeliga@gmail.com (A.S.); blazejmeczekalski@yahoo.com (B.M.); 2 Department of Genetics, Faculty of Biological Sciences, University of Valencia, and INCLIVA, Burjassot, 46100 Valencia, Spain; miguel.garcia@uv.es; 3 Department of Cellular Biology, Functional Biology and Physical Anthropology, Faculty of Biological Sciences, University of Valencia, Burjassot, 46100 Valencia, Spain; juan.j.tarin@uv.es; 4 Salus Vitae Women’s Health Clinical Center, 46004 Valencia, Spain

**Keywords:** ovary, aging, fertility, hormones, diminished ovarian reserve, menopausal transition

## Abstract

Ovarian aging is characterized by a gradual decline in both reproductive and endocrine functions, ultimately culminating in the cessation of ovarian activity around the age of 50, when most women experience natural menopause. The decline begins early, as follicular attrition is initiated in utero and continues throughout childhood and reproductive life. Most follicles undergo atresia without progressing through substantial stages of growth. With increasing age, a pronounced reduction occurs in the population of resting follicles within the ovarian reserve, accompanied by a decline in the size of growing follicular cohorts. Around the age of 38, the rate of follicular depletion accelerates, sometimes resulting in diminished ovarian reserve (DOR). The subsequent menopausal transition involves complex, irregular hormonal dynamics, manifesting as increasingly erratic menstrual patterns, primarily driven by fluctuations in circulating estrogens and a rising incidence of anovulatory cycles. In parallel with the progressive depletion of the follicular pool, the serum concentrations of anti-Müllerian hormone (AMH) decline gradually, while reductions in inhibin B levels become more apparent during the late reproductive years. The concomitant decline in both inhibin B and estrogen levels leads to a compensatory rise in circulating follicle-stimulating hormone (FSH) concentrations. Together, these endocrine changes, alongside the eventual exhaustion of the follicular reserve, converge in the onset of menopause, which is defined by the absence of menstruation for twelve consecutive months. The mechanisms contributing to ovarian aging are complex and multifactorial, involving both the oocyte and the somatic cells within the follicular microenvironment. Oxidative stress is thought to play a central role in the age-related decline in oocyte quality, primarily through its harmful effects on mitochondrial DNA integrity and broader aspects of cellular function. Although granulosa cells appear to be relatively more resilient, they are not exempt from age-associated damage, which may impair their hormonal activity and, given their close functional relationship with the oocyte, negatively influence oocyte competence. In addition, histological changes in the ovarian stroma, such as fibrosis and heightened inflammatory responses, are believed to further contribute to the progressive deterioration of ovarian function. A deeper understanding of the biological processes driving ovarian aging has facilitated the development of experimental interventions aimed at extending ovarian functionality. Among these are the autologous transfer of mitochondria and stem cell-based therapies, including the use of exosome-producing cells. Additional approaches involve targeting longevity pathways, such as those modulated by caloric restriction, or employing pharmacological agents with geroprotective properties. While these strategies are supported by compelling experimental data, robust clinical evidence in humans remains limited.

## 1. Introduction

The ovary is a reproductive and endocrine organ with a finite functional lifespan, typically ceasing activity around the age of 50, when most women undergo natural menopause [1]. This loss of function results from the depletion of ovarian follicles, which are the basic structural and functional units responsible for both hormonal production and oocyte release.

Follicles are formed during fetal development as structures consisting of a single oocyte surrounded by flattened granulosa cells, collectively termed primordial follicles. Within these structures, oocytes enter meiosis but arrest at the diplotene stage of the first meiotic prophase [2]. Over time, subsets of primordial follicles are recruited into growth phases, wherein the oocyte matures in parallel with granulosa cell proliferation and differentiation. These cohorts progressively decline in size as they advance in maturation, with only a single follicle ultimately attaining full functionality and releasing a mature oocyte at ovulation.

**Figure 1:**
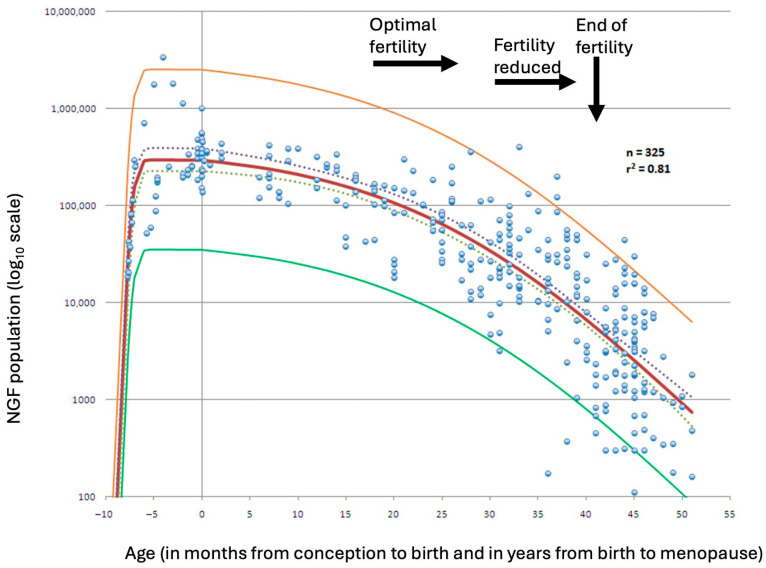
Best-fitting model for the establishment of the nongrowing follicle (NGF) population after conception (red line), based on a dataset of 325 observations. The model is shown with its 95% prediction limits (yellow and green lines) and 95% confidence intervals (dashed lines). Adapted from Wallace & Kelsey [9]. Permission conveyed from Creative Commons (https://creativecommons.org/licenses/by/4.0/, accessed on 8 August 2025).

Follicular depletion begins in utero and continues throughout life, including during childhood. Prior to puberty, ovulation does not occur, and most follicles undergo atresia, that is, degeneration, without initiating growth. Even after puberty, the majority of developing follicles are destined for atresia, as typically only one will complete maturation and ovulate. With increasing age, the continued loss of both dormant and growing follicles leads to a progressive decline in the ovarian reserve. The rate of decline in ovarian follicle number accelerates once the reserve falls to approximately 25,000 follicles, a threshold typically reached at around 37.5 years of age [3] (Figure 1). Although some experimental studies have shown evidence of follicular regeneration in rodents [4,5] and via pluripotent stem cells [6], any such regenerative capacity in humans appears negligible. As hormonal production wanes, menstrual irregularities may occur in some women, although this is not universally observed.

The decline in ovarian function does not affect fertility and hormone production symmetrically. Fertility decreases markedly and ceases approximately a decade before menopause, as supported by population-level data showing that the median age of the last childbirth is around 40 years [7]. In contrast, estrogen production remains clinically adequate until more advanced stages of follicular depletion. During ovulatory cycles, estrogen is produced primarily by the dominant follicle and, to a much lesser extent, by antral follicles in the contemporaneously growing cohorts. As the number of growing follicles declines, estrogen production also decreases. In anovulatory cycles, progesterone is absent. Once the estrogen levels fall below a critical threshold, a state of hypoestrogenic amenorrhea ensues. Menopause is defined by the absence of menstrual bleeding for one year or is classified as premature ovarian insufficiency (POI) if it occurs before the age of 40 [8].

## 2. Clinical Steps of Ovarian Aging

The key characteristics of ovarian aging are outlined in the *Stages of Reproductive Aging Workshop* (STRAW), a consensus statement developed by a panel of experts and first published in 2001 [10]. A subsequent revision, STRAW+10, was released in 2012 [11] with the aim of refining the original criteria, assessing their applicability to specific subpopulations of women and identifying persisting gaps in knowledge and research.

**Figure 2:**
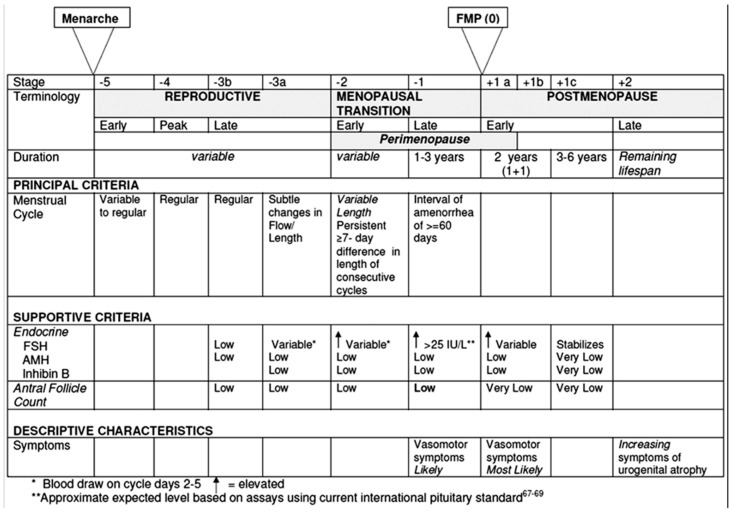
Ovarian function across the stages defined by the *Stages of the Reproductive Aging Workshop +10* (STRAW +10). From Harlow et al. [11]. Reprinted with permission from Elsevier. Permission granted on the 6 May 2025. Copyright conveyed through Copyright Clearance Center.

STRAW categorizes the natural history of ovarian function into three major phases: the reproductive phase, the menopausal transition, and postmenopause. In the STRAW+10 model, each of these phases is further subdivided into stages, totaling ten: four in the reproductive phase, two in the menopausal transition, and four in postmenopause (Figure 2). The primary criteria used to distinguish these stages are based on observable features of the menstrual cycle. Additional supportive criteria include hormonal assays, imaging findings, such as the antral follicle count (AFC), and the presence of symptoms, which are considered descriptive and applied when relevant.

Fertility aspects were not explicitly addressed in STRAW+10, although a progressively decline in fertility is observed with age along the reproductive phase [7]. This occurs in the presence of mostly regular menstrual cycles. The earliest of the four stages in the reproductive phase, following menarche, is marked by some irregularity, whereas the final, fourth stage, is distinguished by subtle alterations in cycle length and menstrual flow. Endocrine changes also accompany this phase. The levels of follicle-stimulating hormone (FSH) begin to rise modestly in the fourth stage, while anti-Müllerian hormone (AMH) levels progressively decline throughout the reproductive years.

### 2.1. Decline of Fertility

The gradual decline in fertility with advancing age has been well documented in couples not using contraception [12]. This reduction in reproductive potential affects all stages of the reproductive process, including fertilization, implantation, and miscarriage risk. Although the underlying causes of this decline remain uncertain, it becomes clinically significant from approximately the age of 38 [3].

#### 2.1.1. Diminished Ovarian Reserve

There is considerable inter-individual variability in the age at which women reach a stage of significant follicular depletion. Diminished ovarian reserve (DOR) is a condition that, in strict terms, refers to a markedly reduced follicular pool resulting from the progressive attrition of ovarian follicles [13,14,15]. Conceptually, it may be understood as representing the tail of a Gaussian distribution of the number of follicles at a specific age. In practice, however, applying the DOR concept remains challenging, as it is inconsistently used to describe diminished oocyte quantity but also reduced oocyte quality or even a broader decline in reproductive potential [14]. Owing to inconsistent definitions and diagnostic criteria, estimates of DOR prevalence vary widely; a prevalence range of approximately 10–26% has been proposed among women seeking fertility treatment [16,17].

The association between DOR and reduced oocyte quality remains a matter of scientific contention. This concomitance is plausibly attributable to shared pathogenic pathways that not only expedite the depletion of the follicular pool but also compromise oocyte integrity, thereby diminishing the overall reproductive competence. The precise nature of these pathways is incompletely delineated and almost certainly multifactorial. Candidate determinants encompass genetic variants implicated in ovarian ageing [16], sustained or acute exposure to specific environmental toxicants [18], and iatrogenic factors, including surgical interventions, such as those performed for endometriosis [19], and cytotoxic chemotherapy [20]. The convergent effects of these influences may underlie the recognized value of DOR as a clinically meaningful surrogate marker for diminished reproductive potential.

#### 2.1.2. Tests of Ovarian Reserve

Efforts to identify DOR have focused on the development of ovarian reserve tests, which aim to estimate the remaining follicular pool within the ovary. Owing to the often-parallel decline in both follicle quantity and quality, these tests are frequently employed as indirect indicators of the fertility potential. However, it is important to underscore that the currently available biomarkers are designed to reflect the ovarian reserve, not fertility per se. As noted in the preceding section, this conceptual distinction remains the focus of considerable scholarly debate [21,22].

The STRAW+10 classification system includes the primary biomarkers of ovarian reserve: AMH, inhibin B, FSH, and AFC.

##### AMH

AMH belongs to the transforming growth factor-β (TGF-β) superfamily and plays a critical role in the differentiation and functional development of reproductive tissues [23].

Within the ovary, it is secreted by granulosa cells of pre-antral and small antral follicles [24]. Because circulating AMH directly reflects the number of producing granulosa cells, its serum concentration serves as a reliable proxy for the size of the cohort of follicles engaged in each wave of folliculogenesis.

Folliculogenesis extends over several months, and overlapping follicular cohorts therefore create a near steady-state output of AMH. This baseline production mirrors the aggregate activity of growing follicles even before they enlarge to the antral stage. Extensive clinical studies substantiate AMH’s value as a biomarker of ovarian reserve [24,25].

The serum AMH levels decline progressively with age, a pattern that closely parallels the shrinkage of the resting follicle pool. Histological analyses and ultrasonographic assessments further corroborate the link between the size of growing follicular cohorts and that of the residual primordial population [26,27]. Although the intra-ovarian mechanisms that govern recruitment remain incompletely defined, it is generally accepted that a smaller primordial pool yields smaller sequential cohorts.

AMH itself inhibits the recruitment of primordial follicles into the growth pathway [28]. Consequently, its age-dependent decline may loosen this inhibitory brake, accelerating follicular depletion and establishing a self-reinforcing feedback loop that further erodes the ovarian reserve.

##### Inhibin B

**Figure 3:**
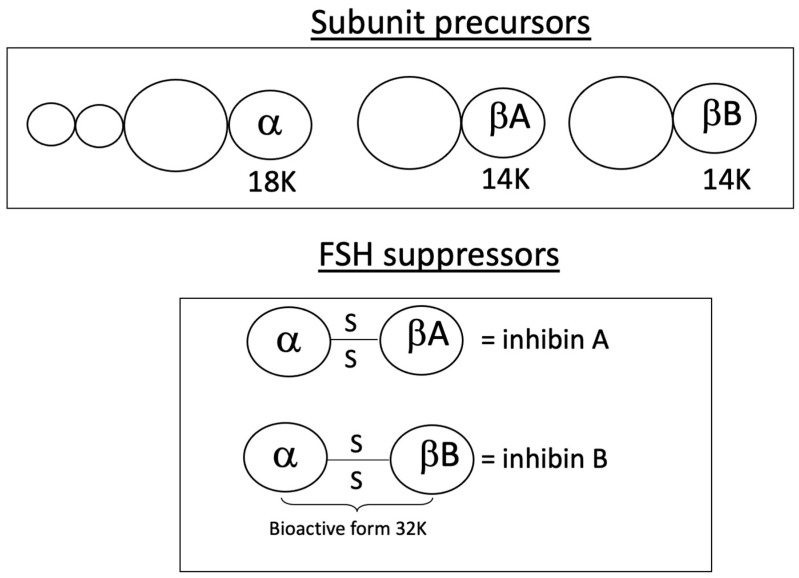
Molecular structure of inhibins A and B. The α-subunit is linked to each of the β-subunits by a di-sulfide bond.

Inhibin B, a dimeric peptide produced by granulosa cells, consists of two subunits: a common α-subunit and distinct β-subunits (βA for inhibin A, βB for inhibin B) (Figure 3). Both forms are stimulated by FSH and modulated by local autocrine and paracrine signals [29,30].

Inhibin B is mainly secreted during the follicular phase by small antral follicles, which makes it a potential biomarker for ovarian reserve. However, its clinical applicability has been regarded as limited due to substantial intra- and inter-cycle variability, although this interpretation has been questioned in some studies [31]. Nevertheless, inhibin B maintains a selective inhibitory effect on FSH. While the intrafollicular concentrations of inhibin B appear to remain relatively unaffected by age [32], its circulating levels decline, most likely due to the reduced number of follicles within each cohort.

##### Other Markers

FSH is a heterodimeric glycoprotein composed of α and β subunits, synthesized in gonadotrope cells of the anterior pituitary. FSH exerts complex and diverse effects, influencing both reproductive and metabolic processes [33]. Its synthesis and release are indirectly regulated by various molecules, including estrogens and ovarian peptides, such as inhibin B, inhibin A, and activin. A decline in inhibin B at the end of the reproductive phase (stage −3a) correlates with an increase in FSH, which becomes evident in the early days of the menstrual cycle. This rise in FSH has historically been used as an indicator of reduced follicle cohort size, particularly before the advent of reliable AMH measurement techniques [34].

AFC is a parameter assessed through vaginal ultrasound. Similar to AMH, which reflects the size of the follicular cohort, AFC provides an evaluation of the cohort at later stages, when the antral follicles have reached a size sufficient for visualization via endovaginal ultrasound. The correlation between AMH-producing follicles and those at later developmental stages, characterized by a well-developed antrum, underpins the utility of this marker [35].

### 2.2. Endocrine Decline

#### 2.2.1. Menopausal Transition

At a certain point after the loss of fertility, the hormonal output deteriorates sufficiently to cause noticeable changes in the menstrual cycle. The most distinct alterations occur during the menopausal transition, which is divided into early and late stages. Changes in the early stage should present differences of ≥7 days in the length of the menstrual cycle, while in the late stage the amenorrhea intervals should reach ≥60 days. The duration of the early stage is variable, while the late stage typically lasts 1–3 years [10].

In the early stage, clinical disturbances consist of mild menstrual irregularities and/or hormonally driven symptoms. The endocrine profile is marked by a progressive increase in FSH levels at the start of the cycle, paralleling a continuous decline in both AMH and inhibin B, the latter contributing to the aforementioned increase in FSH. Additionally, a reduced corpus luteum activity, with decreased secretion of progesterone and inhibin A, further contributes to the early rise in FSH [36]. This early increase in FSH, which may even occur towards the end of the previous cycle’s luteal phase, prompts the rescue of antral follicles within the cohort to complete selection at an earlier stage. Consequently, the follicular phase shortens, reducing the number of days until ovulation. The cycle remains regular, albeit shorter.

The progressive reduction in the number of follicles in growing cohorts is not regular. Therefore, this heterogeneity means that the combination of elevated FSH levels and a greater number of follicles in the cohort results in hyper-estrogenic cycles that can intermingle irregularly with others with lower estrogen levels. Symptoms of both phenotypes may fluctuate accordingly, with vasomotor symptoms (VMSs) affecting a significant percentage of women, particularly in the late stage. These erratic cycles ultimately give way to more frequent anovulation during the late stage of the menopausal transition. Anovulation, when occurring alongside sufficient estrogen levels, can lead to irregular and sometimes severe bleeding, which may necessitate pharmacological or surgical intervention [37]. The most common anovulation phenotype, however, is hypoestrogenic, often presenting with amenorrhea [38].

Hormonal changes in the late menopausal transition phase include a further increase in FSH, alongside persistently low levels of AMH and inhibin B, with a concomitant reduction in antral follicle count. The transition culminates in menopause, defined as the date of the final menstrual period.

#### 2.2.2. Post-Menopause

Post-menopause is characterized by a consistent state of hypo-estrogenic amenorrhea, which requires a duration of 12 months to consolidate the diagnosis. This phase is divided into four stages, with each of the first two stages (+1a and +1b) lasting one year. VMSs are common, affecting up to 80% of women in population studies [39]. FSH levels remain elevated, with unstable values in +1a and stable levels in +1b. Concurrently, elevated levels of luteinizing hormone (LH) are observed. The levels of both AMH and inhibins A and B are very low or undetectable, and antral follicle count is minimal. Androgens are barely affected during the menopausal transition and postmenopause, since their ovarian source consists mainly of stromal cells. In fact, androgens experience a slow and persistent decline from the early reproductive years onwards, primarily due to the age-related decline in the adrenal supply [40].

The third stage of post-menopause, +1c, lasts 3–6 years, followed by the fourth stage, +2, which continues for the remainder of the lifespan. During these stages, hormonal levels and antral follicle count remain unchanged, and VMSs may decrease, although this pattern varies considerably.

## 3. Oxidative Stress and Ovarian Aging

The observed asymmetry in the decline of fertility and endocrine function in the ovary has led researchers to propose distinct functions for the two main cell types within the follicle, i.e., somatic cells and the oocyte. Since fertility declines while the ovary still contains enough follicles to sustain ovulatory menstrual cycles for several years, the concept of quality, as opposed to quantity, has been consolidated as a critical factor.

A long-standing premise has been that much of the fecundity of the follicle and the subsequent stages of reproductive success rests on the functional state of the oocyte. This hypothesis arises from the apparent divergence in the functional trajectories of somatic cells and oocytes in aging follicles. While the precise mechanisms behind this decline remain unclear, factors such as follicular activation dynamics and prolonged ovarian storage are believed to play a role.

### 3.1. Oocyte

The oocyte is a large, metabolically active cell enriched with organelles required to sustain early embryogenesis. Following colonization of the gonadal ridge, each oocyte becomes enveloped by a single layer of flattened granulosa cells, forming the primordial follicle. The definitive oocyte pool is established through oogonial proliferation during fetal life, a process that ends by 16–20 weeks of gestation [41]. At its peak, the human ovary contains approximately 6–7 million oocytes, and, despite the ongoing debate regarding germ-line stem cells in adult ovaries [42,43,44], there is broad consensus that this number does not increase postnatally.

A major hypothesis posits that progressive oocyte dysfunction is driven by oxidative stress [45]. During mitochondrial electron transport, electron leakage generates reactive oxygen species (ROS) that damage proximate macromolecules, including mitochondrial DNA (mtDNA), lipids, and proteins [46]. This mechanism is thought to underlie oocyte senescence, as the oocyte contains an abundance of mitochondria to meet the high energy demands of early embryonic development [47]. A robust body of evidence indicates an inverse relationship between both quantitative and qualitative mtDNA parameters in oocytes and advancing age [48]. The relatively weak antioxidant defenses of aging oocytes likely exacerbate mitochondrial damage, rendering the cell particularly vulnerable.

### 3.2. Granulosa Cells

Increased oxidative stress has also been shown to affect granulosa cells [49]. The reason why granulosa cells exhibit greater resilience to ROS-induced damage compared to oocytes remains unclear, although their higher numbers and capacity for replication may provide a redundancy advantage, helping to buffer the oxidative damage to some extent. Nevertheless, like oocytes, granulosa cells accumulate ROS over time, which target mitochondrial membranes, leading to reduced respiratory efficiency and diminished adenosine triphosphate (ATP) production. As with the oocyte, mtDNA dysfunction in granulosa cells may manifest both quantitatively, such as through mtDNA deletions, and qualitatively through oxidative damage, making the inverse correlation between mtDNA integrity and age a prominent feature of granulosa cell aging [50]. Indeed, both cellular and mitochondrial membranes in granulosa cells deteriorate with age. However, the specific deficiencies associated with aging are not fully understood. A diminished ability to repair DNA has been observed in primates, although its correspondence in humans remains less clear [49].

The aging of granulosa cells may potentially affect both steroidogenesis, thereby altering estrogens output, and the health of the oocyte, given the interconnectedness of these cell types [51]. The proliferation of granulosa cells in growing follicles, along with their substantial presence both in the periphery and in the cumulus oophorus, may help mitigate granulosa-dependent damage, thereby preserving hormone production and contributing to oocyte health.

### 3.3. Ovarian Stroma

Age-associated structural remodeling of the ovarian stroma further illustrates the pervasive impact of oxidative and inflammatory stress. Hallmark changes include progressive fibrosis, stromal expansion, arteriolar obliteration and alterations in cortical and medullary volumes [52]. Fibrosis is characterized by a gradual replacement of elastin with collagen and is accompanied by a pro-inflammatory microenvironment, typified in murine models by increased macrophage and lymphocyte infiltration and elevated cytokine levels. While the precise physiological consequences of these histological changes remain to be defined, accumulating evidence suggests that stromal remodeling may perturb follicular dynamics and compound functional decline [53].

## 4. Other Mechanisms in Ovarian Aging

**Figure 4:**
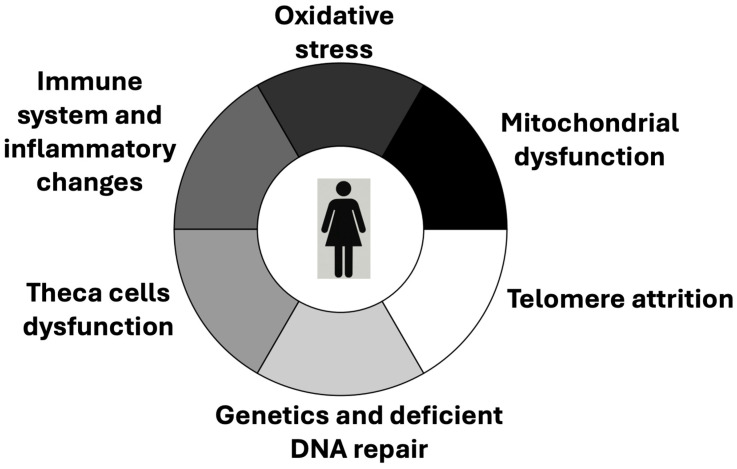
Mechanisms that are attributed a role in the ovarian aging process.

Beyond the central focus on oocytes and granulosa cells, extensive research accumulated over recent years demonstrates that the ovary is a complex gland composed of a diverse array of cellular populations. These include stromal cells, endothelial cells, immune cells, smooth-muscle cells, and, potentially, pluripotent stem cells [50]. These various cell types form an intricate network that, directly or indirectly, interacts with follicular components and modulates their function. In addition to the still-debated presence of pluripotent stem cells, this cellular network also includes thecal cells, derived from stromal precursors, and epithelial cells of the ovarian surface [50]. Together, these elements orchestrate a range of mechanisms implicated in ovarian aging (Figure 4). Thus, the ageing of the oocyte–granulosa complex must be viewed within a broader context of inter-dependent cellular dynamics. While our understanding of this multifaceted network remains incomplete, its study is of substantial scientific and clinical interest. The breadth of recent developments on this topic exceeds the scope of the present discussion; however, several salient points can be highlighted.

### 4.1. Telomere Attrition

Oxidative stress, previously discussed, contributes to telomere attrition, which has been linked to oocyte ageing [54,55]. This finding builds on the well-established association between declining telomerase activity and follicular depletion with advancing age in humans [56]. Clinical evidence further supports this association: reduced telomere length and diminished telomerase activity have been observed in women with POI [57,58,59]. In murine models, telomere length also predicts oocyte quality [60] and has even been proposed as a potential biomarker [61].

### 4.2. Genetics

Genetic mechanisms have also been implicated in ovarian ageing, particularly through compromised DNA repair pathways. Age-related accumulation of double-strand breaks and impaired DNA damage response mechanisms may constitute a key biological axis in ovarian senescence, with a preferential impact on the oocyte [62,63]. This mechanism is especially relevant in women who carry heterozygous pathogenic variants in BRCA1, BRCA2, MCM8, or MCM9, in whom studies report lower AMH concentrations, reduced ovarian reserve, and a tendency toward earlier menopause [62,63,64,65]. In consistence with these data, genome-wide association studies have identified numerous loci mainly related to double-strand break repair, but also immunology [66].

### 4.3. Thecal Cells

Other ovarian cell types, including thecal cells, differentiated from mesenchymal precursors, are increasingly recognized as regulators of ovarian senescence that can secondarily influence oocyte and granulosa cell ageing [67]. Thecal cells organize into concentric layers around growing follicles and secrete androgens that are aromatized by granulosa cells to estrogens, while also producing extracellular-matrix components that function as a selective barrier. Their putative contribution to ovarian aging may involve the release of pro-apoptotic proteins that are transferred to granulosa cells, although this hypothesis remains insufficiently explored [68].

### 4.4. Immune System

The immune system also plays a multifaceted role in regulating the follicular pool, contributing to follicular growth, ovulation, atresia, and the clearance of senescent or damaged cells within the ovarian cortex [69]. The regulatory potential of immune cells in ovarian function is underscored by autoimmune conditions that lead to lymphocytic oophoritis and premature follicular depletion. Aging induces profound changes in ovarian immune dynamics, shifting the balance toward adaptive immunity and away from innate immune responses. Moreover, the number of immune cells in the aging ovary doubles, with a marked increase in lymphocyte populations [70], contributing to a pro-inflammatory milieu. Despite this numerical increase, the functional competence of the immune system declines with age, promoting fibrotic remodeling of the ovarian stroma. The immunological dimension of ovarian aging remains an underexplored yet promising area of research.

## 5. Reversal of Ovarian Aging, from Bench to Bedside

The potential to extend the functional lifespan of the ovary has garnered increasing scientific interest, not only due to its health implications but also because of its broader societal relevance. The growing global burden of infertility, especially pronounced in high-income countries, has catalyzed extensive research into fertility preservation strategies. In clinical practice, cryopreservation of oocytes and ovarian tissue fragments has been implemented for several years [71]. More recently, research efforts have shifted toward exploring the possibility of directly modulating the molecular and cellular mechanisms underpinning ovarian aging. The convergence of deeper mechanistic insights and rapid technological advances is giving rise to a wide spectrum of potential interventions. Nonetheless, most of these approaches remain at the experimental stage. Among the most prominent and promising strategies are stem cell therapy, mitochondrial transfer, and caloric restriction-based interventions [72].

### 5.1. Stem Cells

The growing application of stem cell technologies in reproductive medicine has led to the identification of stem cell populations in various human tissues. Particularly appealing is the use of autologous sources, which circumvent immune rejection and ethical complications. Many stem cell types have been derived from conception-related tissues, such as the amniotic membrane, umbilical cord, and placenta, as well as from tissues with high regenerative turnover, including menstrual blood-derived endometrial cells and human endothelial progenitor cells. A notable example is the intraovarian injection of autologous bone marrow-derived stem cells, a technique referred to as autologous stem cell ovarian transplantation (ASCOT). When combined with platelet-rich plasma (PRP), this approach yielded promising results in a pilot clinical study, including increases in AMH levels and AFC [73]. Subsequent validation in a murine model further reinforced its potential [74].

PRP itself, a biologically active concentrate rich in platelets, cytokines, and growth factors, has shown some efficacy in improving markers of ovarian reserve [75] and reactivating ovarian function in cases of poor ovarian response, POI, or even natural menopause [76]. However, clinical experience with this approach remains limited [77].

An emerging area of innovation involves the use of exosomes, nano-sized vesicles secreted by stem cells, now recognized as key mediators of their regenerative effects [78]. Remarkably, recent advances in bioengineering have enabled the genetic modification of exosomes to deliver targeted therapeutic payloads. For instance, a recent study engineered exosomes derived from mesenchymal stem cells to overexpress programmed cell death protein 1 (PD-1) and galectin-9, two immunomodulatory molecules that can attenuate the autoimmune activity of T lymphocytes. This intervention protected ovarian cells from immune-mediated damage in a murine model of POI [79].

### 5.2. Mitochondrial Transfer

Mitochondrial transfer has also attracted attention, stemming from the critical role of mitochondria in supplying energy to the metabolically demanding ovarian follicle. Experimental studies in mice have consistently demonstrated improved reproductive outcomes following the microinjection of autologous mitochondria [80].

In humans, mitochondria from various autologous sources have been explored, with preliminary findings suggesting potential efficacy comparable to that observed in animal models [81]. Nevertheless, this technique remains largely unregulated and is offered predominantly in private clinics. In contrast, the transfer of donor-derived mitochondria, also known as mitochondrial replacement therapy, has been developed for the treatment of mitochondrial diseases. However, this approach remains highly restricted due to ethical concerns and is currently approved in only a limited number of countries [82].

### 5.3. Other Therapeutic Options

Caloric restriction has long been associated with increased lifespan in various animal models, including non-human primates, without inducing malnutrition [83]. Substantial molecular evidence supports its anti-aging effects, and several pathways linking caloric restriction to the delay of senescence have been identified [83,84]. On this basis, pharmacological mimetics such as rapamycin are being explored for their potential to replicate these benefits [85].

Pharmacological interventions aimed at delaying ovarian aging are also under investigation. Among these, metformin has emerged as a candidate due to its immunomodulatory and antifibrotic properties. Preclinical studies have demonstrated that metformin can modulate immune cell populations and fibroblasts, thereby preventing age-associated ovarian fibrosis in rodent models [86]. Nonetheless, the current body of evidence remains preliminary, underscoring the need for further rigorous investigation [87].

## 6. Conclusions

The decline in ovarian function follows distinct timelines for the two primary functions of the ovary: fertility and hormonal production. In both cases, the decline is often gradual, with intermittent periods of relative stability. This means that, while overall dysfunction progresses, periods of normality may still occur, although they become less frequent with advancing age. Clinically, this progressive decline has been conceptualized as DOR for fertility and menopausal transition for hormonal function. The precise mechanisms behind these age-related functional deficiencies remain elusive, but oxidative stress has been identified as a key contributor, based on data from various experimental models and human studies. While these mechanisms aim to reflect the effects of aging on the later stages of ovarian function, it is likely that other factors, such as genetic dysfunction in genes involved in the regulation of ovarian aging, epigenetic influences, and environmental factors including surgical interventions may also modulate this age-dependent deterioration. Moreover, advances in the understanding of ovarian aging have paved the way for novel interventions aimed at prolonging ovarian function. Although still largely experimental, preliminary evidence in humans supports the potential use of autologous stem cells, which are also recognized as a source of exosomes, a promising therapeutic avenue. The autologous transfer of mitochondria has likewise attracted interest, due to its demonstrated efficacy in animal models and anecdotal human cases, although its legal status remains uncertain. Other proposed strategies, such as caloric restriction and certain pharmacologic or hormonal agents, possess biological plausibility and experimental support; however, robust clinical data in humans are still lacking.
